# Optimizing P3HT/PCBM-Based Organic Photodetector Performance: Insights from SCAPS 1D Simulation Studies

**DOI:** 10.3390/nano14131146

**Published:** 2024-07-04

**Authors:** Ahmet Sait Alali, Murat Oduncuoglu, Farid Touati

**Affiliations:** 1 Department of Physics, Yıldız Technical University, 34349 Istanbul, Turkey; ahmet.alali@std.yildiz.edu.tr; 2 Department of Electrical Engineering, College of Engineering, Qatar University, Doha 2713, Qatar; touatif@qu.edu.qa

**Keywords:** SCAPS 1D, P3HT, PCBM, photodetector, optimization, responsivity

## Abstract

Organic electronics have great potential due to their flexible structure, high performance, and their ability to build effective and low-cost photodetectors. We investigated the parameters of the P3HT and PCBM layers for device performance and optimization. SCAPS-1D simulations were employed to optimize the thicknesses of the P3HT and PCBM layers, investigate the effects of shallow doping in the P3HT layer, and assess the influence of the back contact electrode’s work function on device performance. Furthermore, this study explored the impact of interface defect layer density on the characteristics of the device. Through systematic analyses, the optimal parameters for enhancing device responsivity were identified. The findings indicate that a P3HT layer thickness of 1200 nm, a PCBM layer thickness of 20 nm, and a back contact electrode with a work function of 4.9 eV achieve the highest responsivity. Notably, at a bias of −0.5 V, the responsivity exceeds 0.4 A/W within the wavelength range of 450 nm to 630 nm. These optimized parameters underscore the significant potential of the developed device as an organic photodetector, particularly for visible light detection.

## 1. Introduction

Organic photodetectors (OPDs) made from organic polymers and molecules offer several advantages that make them highly useful for various applications. These devices include superior photodetection performance, low production costs, fabrication versatility, mechanical flexibility, and the capability for large-area fabrication [1,2]. Additionally, OPDs can exhibit a broad spectral response range, high responsivity, and high response frequency, making them ideal for sensing white and near-infrared light [3]. Furthermore, the carrier blocking layers in OPDs play a crucial role in reducing dark current, enhancing efficiency, and ensuring long-term stability, thus contributing significantly to their overall performance [3]. As a result, these advantages make organic photodetectors promising candidates for next-generation photodetection technologies.

The performance of organic photodetectors (OPDs) is significantly influenced by their responsivity, which is a measure of their ability to convert light into an electrical signal. Limitations in responsivity can adversely affect OPD performance in several ways. Firstly, surface defects and charge trapping in materials can lead to persistent photoconductance, hindering responsivity and overall efficiency in optoelectronic applications [2]. Similarly, the spectral response properties of materials indicate that increased defects can reduce detector responsivity, highlighting the necessity of defect control during fabrication [3]. Secondly, integrating organic and inorganic materials has been explored to enhance OPD responsivity. The incorporating graphene oxide in a hybrid trilayer photodetector structure has demonstrated improved responsivity and a broad bandwidth, crucial for sensing white and near-infrared light [4]. Furthermore, the coexistence of discontinuous single layers and multilayers in 2D materials such as tungsten disulfide (WS2) poses challenges for the large-scale fabrication of high-performance photodetectors [5]. Additionally, the introduction of plasmonic materials, such as Ag nanoparticles, can extend the spectral response range of OPDs. However, careful consideration must be given to the potential increase in dark currents, which can affect the signal-to-noise ratio and thus the responsivity of the device [6].

As a result of advancements in material science and engineering, significant improvements have been achieved in the performance of organic photodetectors (OPDs). However, limitations in responsivity due to factors such as surface defects, material inhomogeneity, and mechanical flexibility remain critical challenges that need to be addressed to further enhance OPD performance [4,7,8]. The development and optimization of photodetector systems often begin with the establishment of a robust mathematical model that accurately captures the underlying physical principles governing their operation. This comprehensive approach, spanning from the material-level properties to the system-level performance, is crucial for ensuring the compatibility of laboratory prototypes with industrial technologies [9,10,11]. In this study, our primary objective was to understand and design the structure of P3HT/PCBM-based (3-Hexylthiophene)/(Phenyl-C61-butyric acid methyl ester) OPDs and optimize their performance using SCAPS-1D simulations developed by the Department of Electronics and Information Systems of the Ghent University (Ghent, Belgium). In accordance with previous studies, the simulated results obtained from SCAPS demonstrate a satisfactory agreement with the experimental findings [12,13]. The focus lies in elucidating the intricate relationships between key parameters, such as the thicknesses of the P3HT and PCBM layers, the shallow doping of the P3HT layer, and the characteristics of the back contact electrode, including its work function. By systematically varying these parameters and analyzing their effects on device performance, we aim to identify the optimal conditions that yield the highest responsivity within the visible light spectrum. This study seeks to contribute to the advancement of organic photodetector technology by providing valuable insights into the design and optimization of these devices for enhanced sensitivity and efficiency. Additionally, the fundamental mechanisms underlying device operation and performance are investigated to improve the capabilities of next-generation organic photodetectors, with broader applications in fields such as optical communication, imaging, and sensing.

## 2. Device Structure and Numerical Simulations

**Figure 1:**
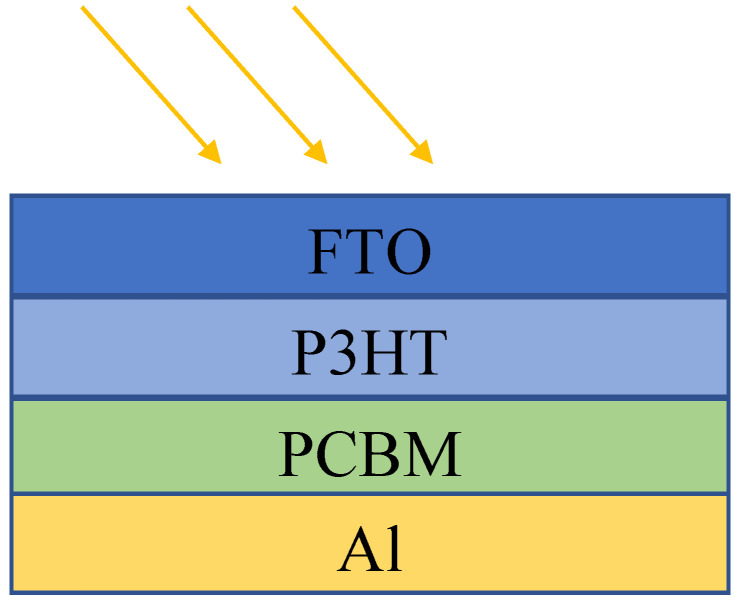
The schematic representation of the OPD used in simulations includes a substrate, an anode, a hole transport layer, a cathode, and an encapsulation layer for protection. Each layer plays a critical role in the device’s performance by facilitating charge transport and protecting the organic materials.

**Figure 2:**
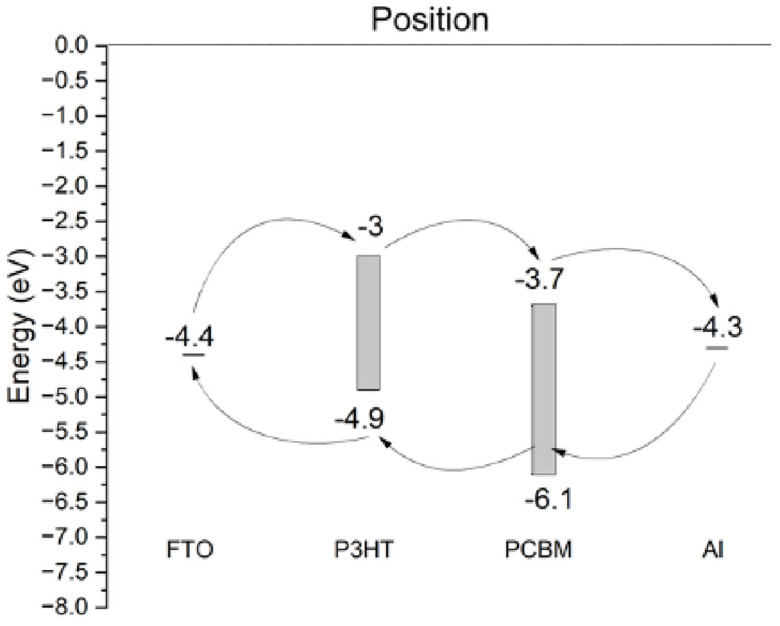
Corresponding schemes of energy-level alignment diagram of the layers used in the simulation. This diagram illustrates the energy levels associated with the FTO substrate, P3HT layer, PCBM layer, and aluminum (Al) electrode, highlighting the alignment and differences in their energy levels, critical for device performance.

The structure of the OPD is designed through the sequential deposition of FTO (fluorine-doped tin oxide), P3HT (poly(3-hexylthiophene)), PCBM (phenyl-C61-butyric acid methyl ester), and aluminum (Al) layers. The FTO layer serves as a transparent conductive substrate, providing a stable foundation for the subsequent layers. Fluorine-doped tin oxide (FTO) and aluminum (Al) are commonly used as electrodes in organic photodetectors (OPDs). FTO is often utilized as a transparent anode due to its high transparency and low sheet resistance, allowing efficient light transmission and charge extraction [14,15]. On the other hand, Al is frequently employed as the cathode in OPDs, contributing to the efficient collection of photogenerated carriers [12,13,16]. The P3HT layer, which comprises poly(3-hexylthiophene), is deposited next and acts as the photoactive material that absorbs incident light and generates charge carriers. Poly(3-hexylthiophene) (P3HT) and phenyl-C61-butyric acid methyl ester (PCBM) are widely used in organic electronics due to their complementary properties that enhance device performance. P3HT, a benchmark semiconducting polymer, is known for its excellent electronic properties, including good charge carrier mobility and stability, making it suitable for various applications, such as photovoltaic cells, photodetectors, and transistors [17,18]. PCBM, a fullerene derivative, acts as an efficient electron acceptor, facilitating effective charge separation and transport when blended with P3HT [19]. The P3HT:PCBM blend exhibits favorable thermal and transport properties, with the energy band gap being dependent on the P3HT:PCBM ratio and the thermal diffusivity and charge carrier lifetime being influenced by the PCBM concentration and illumination intensity [20]. The blend morphology and phase behavior are crucial for device performance, with studies showing that thermal annealing can optimize phase separation and improve efficiency [21]. Additionally, the thicknesses of the active layer and the hole transport layer, as well as the electron mobility, play significant roles in enhancing power conversion efficiency (PCE) [22,23,24]. Furthermore, the stability of the P3HT and PCBM layers can be enhanced by incorporating metal ions, which can be beneficial for flexible electronic devices [25,26]. Adjacent to the P3HT layer is the PCBM layer, which functions as an electron acceptor, facilitating the separation of electron–hole pairs created within the P3HT layer upon photon absorption. Finally, the aluminum electrode is deposited on top of the PCBM layer, serving as the electron-collecting contact. Figure 1 and Figure 2 illustrate a schematic representation of the organic photodetector.

The parameters used in simulations for OPD are given in Table 1. These parameters are taken from given references.

## 3. Results and Discussion

### 3.1. Optimization of the Thickness of the P3HT Layer

The thickness of the donor layer within the photoactive bulk heterojunction significantly affects the performance of organic photodetectors. The traditional studies on organic device structures revealed that varying the active layer thickness from 50 to 300 nm significantly impacts the efficiency of the photodiodes [31]. A high performance was achieved despite the surface roughness and morphology of the organic layers, highlighting the optimization potential of these materials. The thickness of the donor layer influences several key parameters that govern the performance of organic photodetectors. A thicker donor layer has the potential to increase light absorption, thereby enhancing the overall photogenerated current. However, this increased thickness can also introduce higher series resistance, which impedes carrier transport and consequently reduces the fill factor. On the other hand, a thinner donor layer can facilitate more efficient carrier collection due to reduced resistance, but this often comes at the cost of incomplete light absorption, leading to lower photocurrent generation [32,33]. In the analysis of a self-powered photodetector, capable of detecting light without requiring an external power supply, using bulk p-n heterojunctions, the optimal thickness of the layer was determined to be 2.5 µm, demonstrating the importance of layer thickness in performance optimization [32].

**Figure 3:**
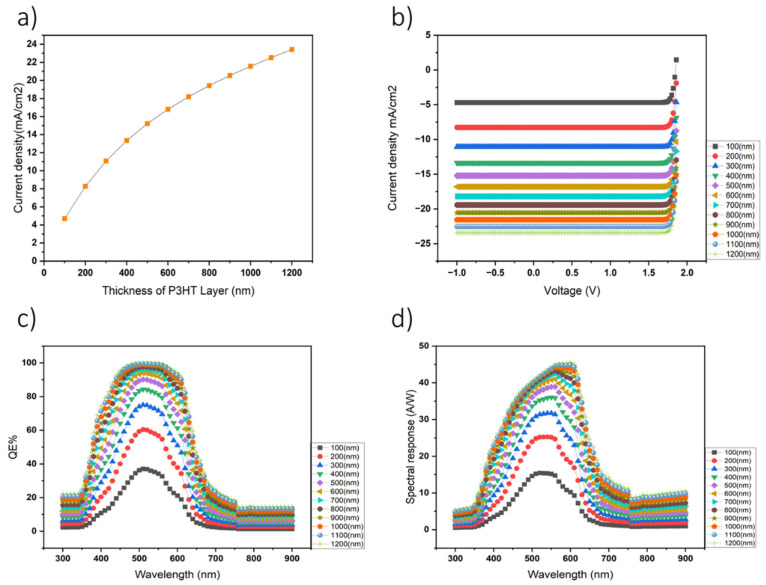
(**a**) The impact of the P3HT layer thickness on photodetector performance. (**b**) Variation in the short-circuit current density (J_sc_). (**c**) The current–voltage (J–V) characteristics of the device. (**d**) The QE of the photodetector. (**e**) The spectral response across different P3HT layer thicknesses. The results in the graphs are based on simulations.

Furthermore, the electrical performance of organic photodetectors has been demonstrated to be highly sensitive to the thickness of the photoactive layer, underscoring the crucial influence of donor layer thickness on photodetector efficiency [34]. After establishing the appropriate layer configurations within the SCAPS simulation environment, a detailed analysis was conducted to evaluate the current density (J) across a range of P3HT layer thicknesses, varying from 50 nm to 1200 nm. This investigation involved generating J–V curves, as well as wavelength-dependent current density profiles for each thickness, enabling a thorough assessment of device performance under different conditions. Additionally, quantum efficiency (QE) and spectral response analyses were performed to elucidate the relationship between wavelength and current density, revealing distinct trends across the spectral range. Notably, our findings highlight a significant correlation between the thickness of the P3HT layer and key device performance metrics (Figure 3).

Specifically, the highest values for current density, QE, and spectral response were consistently achieved with a P3HT layer thickness of 1200 nm. These results highlight the critical role of precise layer thickness optimization in enhancing the performance of organic photodetectors and emphasize the importance of comprehensive parameter optimization to improve the efficiency and effectiveness of these devices in practical applications.

### 3.2. Optimization of the PCBM Layer Thickness

**Figure 4:**
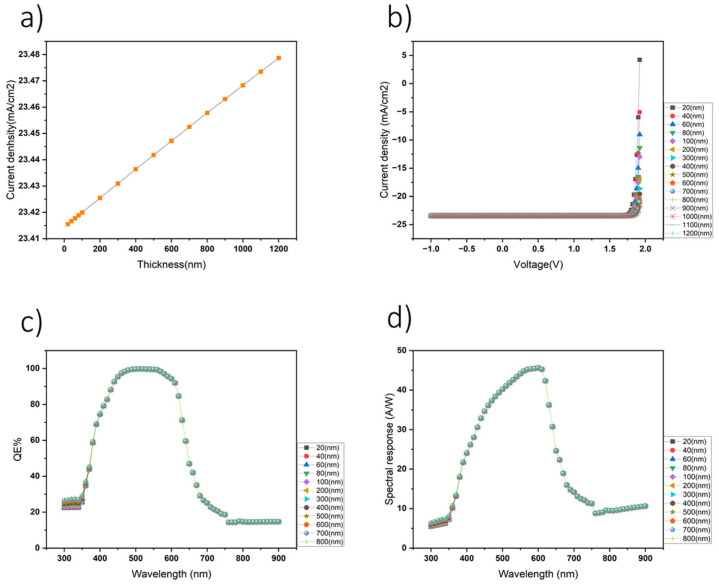
The influence of the PCBM layer thickness on photodetector performance. (**a**) Simulated results depicting the variation in J_sc_. (**b**) The J–V characteristics of the device. (**c**) The QE of the photodetector. (**d**) The spectral response across different PCBM layer thicknesses based on simulations.

The HT layer was determined through comprehensive simulations using SCAPS. Our investigation shifted focus towards assessing the impact of varying the PCBM layer thickness on photodetector performance. Through detailed simulation-based analysis, PCBM layer thicknesses ranging from 20 nm to 1200 nm were systematically tested. The subsequent evaluation involved generating current density–voltage (J–V) characteristics (as shown in Figure 4b) and wavelength-dependent current density profiles for each thickness (Figure 4a). Additionally, the QE (Figure 4c) and spectral response (Figure 4d) analyses were conducted to elucidate the relationship between wavelength and device performance metrics. Remarkably, the simulation results revealed intriguing insights, indicating a notable correlation between the PCBM layer thickness and photodetector performance. Specifically, the photodetector exhibited the highest current density, QE, and spectral response when the PCBM layer was optimized to a thickness of 20 nm. These findings highlight the critical importance of precise layer thickness optimization in enhancing the efficiency and sensitivity of organic photodetectors, offering valuable guidance for further advancements in device design and optimization through simulation-based approaches.

### 3.3. Effect of Varying P3HT Donors on Photodetector Performance

**Figure 5:**
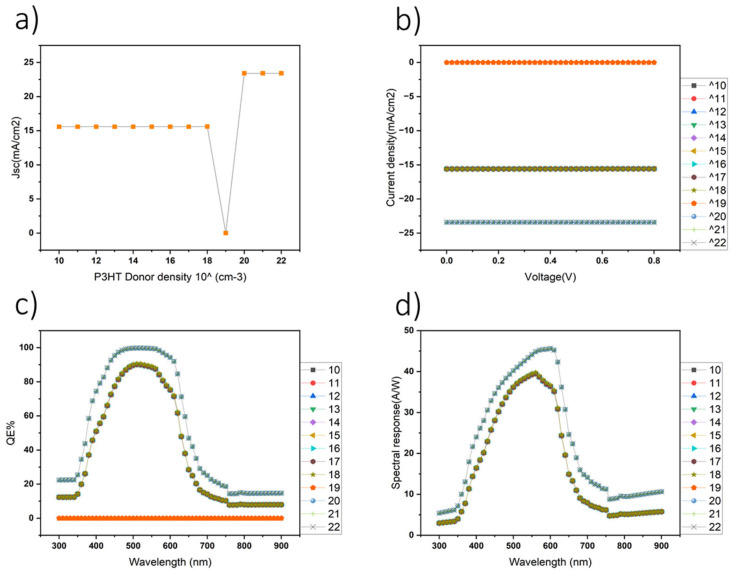
Illustration of the impact of donor concentration on photodetector performance. (**a**) Illustration of the variation in J_sc_ based on simulated results, (**b**) the J–V characteristics of the device, (**c**) the QE of the photodetector, and (**d**) the spectral response across different donor concentrations, as per simulations.

Having identified the optimal thicknesses for both the P3HT and PCBM layers, which exhibited superior performance characteristics, our investigation proceeded to examine the influence of varying the shallow donor density within the P3HT layer on overall device performance. Systematic simulations were conducted across a range of shallow donor densities spanning from 10^11^ to 10^21^ cm^−3^, and their effects on device performance metrics were carefully assessed. Notably, the analysis revealed intriguing trends, as shown in Figure 5a, illustrating the correlation between donor density and current density. The optimal device performance was observed at a shallow donor density of 10^20^, which exhibited the highest current density. Moreover, QE analysis of the optimal donor density (shown in Figure 5c) further corroborated these findings, highlighting the pronounced impact of donor density on device performance.

### 3.4. Effect of the Metal Electrode Work Function

**Figure 6:**
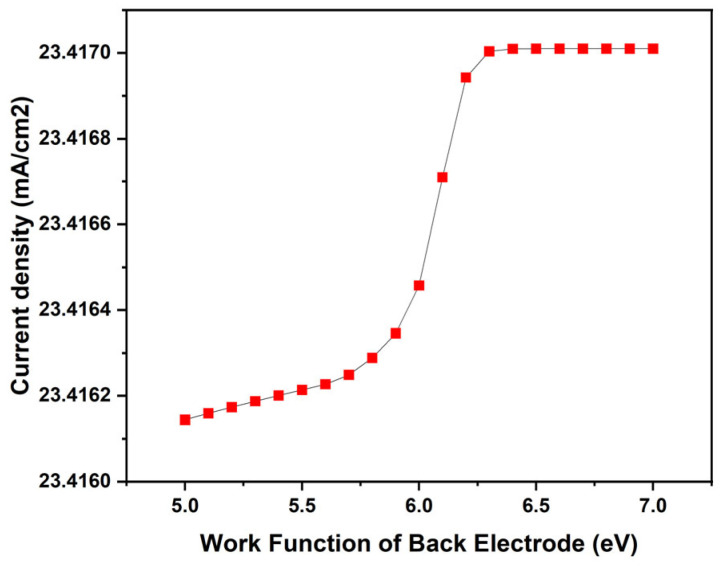
This figure illustrates the variation in short-circuit current density with respect to the metal work function based on simulations.

Metal electrodes, such as Al nanoparticles and nanostructured metal electrodes, significantly impact the responsivity of photodetectors. Added nanoparticles on films enhance the photoresponse by augmenting light scattering and strengthening surface plasmon resonance (SPR) [35]. Similarly, increasing the responsivity and detectivity improved the performance of photodetectors [36]. On the other hand, increased vacancy defects negatively affect carrier recombination, leading to a decrease in the responsivity of detectors [37]. Additionally, nanostructured metal electrodes enable the generation of direct electrical readouts, impacting the device’s optical and electrical response based on the incidence angle and polarization [38]. Metal electrodes play a crucial role in enhancing the performance of photodetectors through various mechanisms. In this study, we analyzed the effect of the work function of the back electrode on the current density of the device. The work function was systematically varied across a spectrum ranging from 5.0 to 7.0 eV. The choice of the back electrode material aligns with its widespread application in electronic devices due to its favorable electrical conductivity and compatibility with device fabrication processes. The results, shown in Figure 6, illustrate the impact of different work function values on device performance metrics.

This comprehensive analysis enables a thorough examination of the relationship between the work function of the back electrode and the overall efficiency and efficacy of the photodetector. These findings provide valuable insights into the optimization of device parameters for enhanced performance and functionality, facilitating advancements in the field of optoelectronics. Within the analyzed work function range of 5.0 to 7.0 eV, we observed a notable trend in the current density of our photodetectors. As the work function increased from 5.0 eV to approximately 6.2 eV, there was a gradual increase in current density, which then plateaued at higher work function values.

This trend can be attributed to the changes in the energy alignment at the electrode interface, which influences the charge carrier injection and extraction processes. The initial increase in the work function leads to a higher rate of electron–hole pair generation and improved charge carrier collection efficiency, as indicated by the rise in current density. Beyond 6.2 eV, the current density stabilizes, suggesting that the optimal work function for maximum device performance has been reached. This analysis underscores the critical importance of the work function in optimizing the performance of the photodetector.

A sharp increase in the short-circuit current density with a variation in the work function can be observed in various contexts. Research on field emission cathodes shows that small variations in the work function impact the transmission coefficient for electron tunneling, affecting the total energy distribution [39]. The findings collectively suggest that work function variation plays a crucial role in modulating device performance metrics such as J_sc_ in various electronic systems.

### 3.5. Effect of the (IDL) Interface Defect Layer

**Figure 7:**
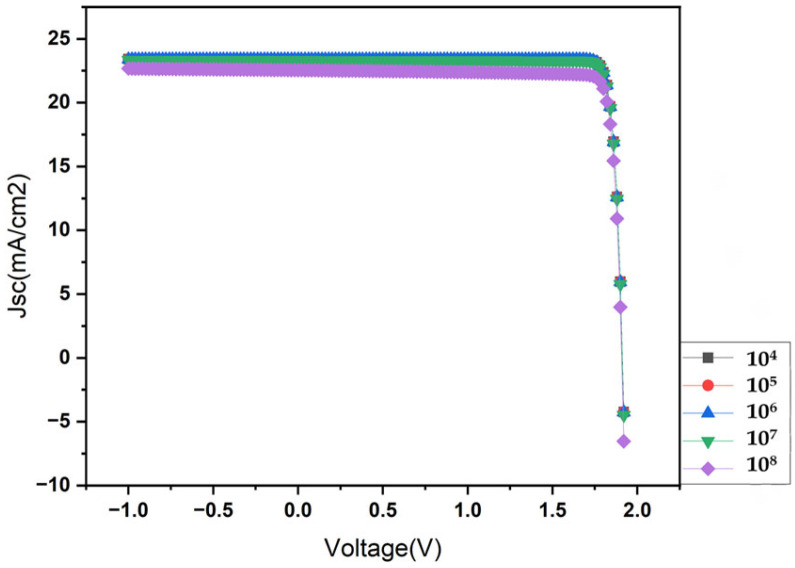
Illustration of the J–V characteristics for the device under investigation, showing the impact of various interface layer defect densities based on simulations.

The IDL plays a crucial role in the performance of photodetectors. Studies have shown that interface engineering techniques significantly impact device characteristics. These approaches have been found to enhance crystallinity, improve charge extraction, reduce dark current, increase responsivity, and extend device stability. IDL engineering plays a critical role in enhancing the efficiency and stability of photodetectors. Figure 7 illustrates the relationship between current density and voltage (J–V) under various interface defect densities (IDLs) ranging from 10^4^ to 10^8^ cm^−2^. A noticeable trend emerges where the current density decreases significantly at the highest IDL of 10^8^ cm^−2^. Conversely, optimal current density values are observed at an IDL of 10^5^ cm^−2^. This finding underscores the critical role of interface defect density in determining device performance, with lower defect densities correlating with improved current density outcomes.

### 3.6. Optimized Device

The responsivity of photodetectors directly influences their sensitivity and overall performance in different environments [4,40]. Responsivity measures the ability of a photodetector to convert incident light into an electrical signal, reflecting its sensitivity to light stimuli. Higher responsivity values indicate a more efficient conversion process, leading to enhanced detection capabilities. For instance, photodetectors with increased responsivity exhibit improved performance metrics, such as higher on/off ratios, detectivities, external quantum efficiencies, and faster response times. Moreover, optimizing responsivity can enable photodetectors to operate effectively in diverse applications, such as optical communication, image detection, environmental monitoring, and optoelectronics, demonstrating their adaptability and reliability in various environments.

The responsivity ℛ represents the sensitivity of the photodetector to the input light. Mathematically, it can be written as

**Figure 8:**
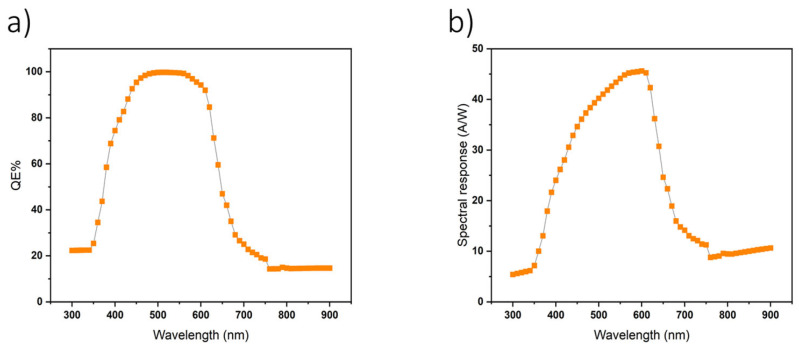
(**a**) QE of the photodetector is shown, and (**b**) displays the spectral response based on simulations.

ℛ = I/P
(1)

where I is the photogenerated current, P is the input optical power, I_p_ is the photocurrent, and p is the incident power. Utilizing the responsivity equation, empirical data was extracted from Figure 8. Our analysis reveals a peak responsivity of 0.456 A/W at a wavelength of 600 nm. Furthermore, within the spectral range spanning from 450 nm to 630 nm, the responsivity consistently surpasses the threshold of 0.4 A/W, indicating robust sensitivity across a broad spectrum of visible light wavelengths. These findings underscore the efficacy of the organic photodetector model for capturing and converting incident light into electrical signals, particularly within the visible range. Consequently, our study highlights the suitability of this organic photodetector configuration for applications requiring the efficient detection and conversion of visible light signals, bolstered by its commendable responsivity performance.

After optimization, the device exhibited notable parameters derived from the calculated IV curve analysis presented in Table 2. The open-circuit voltage (V_oc_) reached 1.912 volts, indicating that the maximum voltage was attainable under illumination conditions. Concurrently, the J_sc_ was 23.415 mA/cm^2^, representing the maximum current output when the device is shorted. Fill factor (FF) achieved an impressive value of 91.101%, indicating the efficiency of the device in utilizing available photogenerated carriers. Moreover, the overall power conversion efficiency (eta) was calculated to be 40.797%, reflecting the device’s ability to convert incident light into electrical energy. Notably, the voltage at the maximum power point (V_MPP_) was determined to be 1.769 volts, while the corresponding current density at the maximum power point (J_MPP_) was 23.064 mA/cm^2^. These detailed parameters, as outlined in Table 2, offer insights into the enhanced performance and efficacy of optimized organic photodetectors device. For a thorough exposition of the device’s architectural specifications, refer to Figure 1.

The relationship between the V_oc_ limits and photodetector sensitivity is crucial for enhancing imaging performance. Research has shown that utilizing an open-circuit voltage pixel (V_ocP_) architecture can significantly improve pixel sensitivity across various spectral ranges, leading to enhanced performance compared to that of conventional pixel topologies [41,42]. In the mid-wave infrared (MWIR) range, the V_ocP_ architecture has demonstrated superior noise equivalent differential temperature (NEDT) compared to conventional reverse-bias (RB) operations, especially considering the actual well capacity of a readout integrated circuit (ROIC) [43]. Additionally, in organic photodetectors (OPDs), operating in the open-circuit voltage regime has proven to be efficient for detecting low light signals, showing a linear and logarithmic relationship between V and irradiance, and achieving high photovoltage responsivity without the need to suppress dark current, thus simplifying the design rules for OPDs [44]. These findings collectively highlight the importance of V_oc_ limits in optimizing photodetector sensitivity for various imaging applications.

**Table 2:** A performance comparison between the proposed OSC and the leading organic solar cells described in the literature.

Ref.	Device Structure	Voc (V)	Jsc (mA/cm²)	FF (%)	PCE (%)
[45]	P3HT/PCBM	0.72	19	63	8.62
[46]	P3HT:PCBM blend with embedded SiO_2_@Ag@SiO_2_ nanoparticles	0.60	16.31	76.6	7.61
[47]	ITO/PEDOT:PSS/P3HT:PC_60_BM/Al	0.66	12.01	59	4.65
[48]	/ITO/PEDOT:PSS/P3HT/P3HT:PCBM/PFN:BR/Al	0.76	22.6	82.33	14.32
This work	FTO/P3HT/PCBM/Al	1.91	23.41	91.10	40.79

The results presented in Table 3 offer a comparative overview of the responsivity values reported in various studies utilizing different materials, alongside the findings from this study after the parameters were optimized. Our investigation revealed that post optimization, our device achieved a significantly enhanced responsivity compared to previous studies, demonstrating the efficacy of the optimized parameters in enhancing device performance.

## 4. Conclusions

In conclusion, our research has yielded significant insights into the performance and optimization of organic photodetectors. Through systematic simulation analyses, we identified key parameters influencing device performance, including the thicknesses of the P3HT and PCBM layers and the shallow donor density within the P3HT layer. These findings underscore the critical role of these parameters in dictating device efficiency and efficacy, providing valuable guidance for the design and optimization of organic photodetectors. Additionally, our investigation into the effect of varying the work function of the metal electrode further enhances our understanding of device behavior and offers avenues for continued improvement in this area. By elucidating these relationships, our study contributes to the advancement of organic optoelectronics, paving the way for the development of high-performance photodetection technologies. Moving forward, further research endeavors could explore additional parameters and configurations to further enhance device performance and expand the applications of organic photodetectors in various fields.

## Figures and Tables

**Table 1:** Physical properties of the materials used in the initial calculation.

Material Properties	FTO [27]	P3HT [28,29]	PCBM [30]
Thickness (nm)	50.00	350.00	80.00
Band gap (eV)	3.500	2.100	2.100
Electron affinity (eV)	4.000	3.500	3.900
Dielectric permittivity	9.000	4.400	3.900
Conduction band effective density of states, n*_c_* (cm^−3^)	2.20 × 10^18^	1.00 × 10^18^	2.20 × 10^19^
Conduction band effective density of states, n*_v_* (cm^−3^)	1.80 × 10^19^	1.00 × 10^18^	2.20 × 10^19^
Electron thermal velocity, V*_e_* (cm/s)	1.00 × 10^7^	1.00 × 10^7^	1.00 × 10^7^
Hole thermal velocity, V*_h_* (cm/s)	1.00 × 10^7^	1.00 × 10^7^	1.00 × 10^7^
Electron mobility, *µ_e_* (cm^2^/Vs)	2.00 × 10^1^	8.00 × 10^−4^	0.001
Hole mobility, *µ_h_* (cm^2^/Vs)	1.00 × 10^7^	8.00 × 10^−4^	0.002
Shallow uniform donor density, n*_D_* (cm^−3^)	2.00 × 10^21^	2.00 × 10^20^	1.00 × 10^16^
Shallow uniform acceptor density, n*_A_* (cm^−3^)	1.00 × 10^20^	1.00 × 10^19^	1.00 × 10^19^
Defect density	1.00 × 10^15^	1.00 × 10^15^	1.00 × 10^18^

**Table 3:** Comparison of device structures between studies. Highlights key differences and advances in this research.

Photoactive Layer	*λ* [nm]	Bias [V]	R [mA/W]	Ref
SnO_2_/CsPbI_3_/CuI	550–600	−0.5	400@580 nm	[49]
PCDTBT and PC_71_ BM	400–700	–5	302@500 nm	[50]
P3HT/PCBM	350–750	−0.5	456@600 nm	This work

## Data Availability

Data are contained within the article.
